# High Serum Carotenoids Are Inversely Associated with Serum Gamma-glutamyl-transferase in Alcohol Drinkers within Normal Liver Function

**DOI:** 10.2188/jea.15.180

**Published:** 2005-09-27

**Authors:** Minoru Sugiura, Mieko Nakamura, Yoshinori Ikoma, Masamichi Yano, Kazunori Ogawa, Hikaru Matsumoto, Masaya Kato, Makoto Ohshima, Akihiko Nagao

**Affiliations:** 1 Department of Citrus Research, National Institute of Fruit Tree Science.; 2 Department of Epidemiology, National Institute for Longevity Sciences.; 3 Food Materials Research Division, National Food Research Institute.

**Keywords:** Carotenoids, gamma-Glutamyltransferase, Alcohol Drinking, Oxidative Stress, Cross-Sectional Studies

## Abstract

BACKGROUND: Many studies have reported that the consumption of alcohol induces the generation of free radicals. Moreover, recent studies suggest that serum gamma-glutamyltransferase (*γ*-GTP) within its normal range might be an early marker of oxidative stress. In this study, we tested the hypothesis that serum antioxidant carotenoids would be inversely associated with serum *γ*-GTP in alcohol drinkers within normal liver function.

METHODS: A total of 266 Japanese men who had received health examination in 2003 participated in the study. The associations of serum *γ*-GTP and serum-carotenoid concentrations stratified by alcohol intake levels were evaluated cross-sectionally. The participants were divided into three groups according to their ethanol intake level (non-drinker, less than 1 g/day; light drinker, 1-25 g/day; and moderate and heavy drinkers, 25+ g/day). The multivariate-adjusted geometric means of the serum *γ*-GTP concentrations in each tertile of the serum-carotenoid concentrations were calculated after adjustment for ethanol intake, age, body mass index, total cholesterol, triacylglycerols, current tobacco use, and habitual exercise.

RESULTS: The serum *γ*-GTP concentrations were significantly high in accordance with the ethanol intake level. In moderate and heavy drinkers, the multivariate-adjusted geometric means of serum *γ*-GTP concentrations were significantly low in accordance with the tertiles of the serum lycopene, *α*-carotene, *β*-carotene, and *β*-cryptoxanthin concentrations.

CONCLUSIONS: The serum antioxidant carotenoids were inversely associated with alcohol-induced increases of serum *γ*-GTP in moderate and heavy drinkers within normal liver function.

It is well known that alcohol induces the generation of free radical species during its metabolism.^1^^-^^3^ Many studies of the antioxidant status of alcohol-induced hepatitis or cirrhosis patients have reported on the measurement of the blood concentrations of antioxidants or markers of oxidative stress, such as *α*-tocopherol, ascorbic acid, carotenoids, or glutathione.^4^^-^^8^ These studies provided support for the hypothesis that antioxidant micronutrients may protect against oxidative stress induced by alcohol consumption.

Although serum gamma-glutamyltransferase ( *γ*-GTP) is not a specific indicator of liver injuries due to alcohol, it is widely used as a screening test for alcohol-induced liver dysfunction.^9^^-^^11^ Recent epidemiologic studies have reported that serum *γ*-GTP within its normal range might be an early marker of oxidative stress.^12^^-^^16^ In these reports, the authors found the inverse associations of serum-carotenoid concentrations with serum *γ*-GTP. However, the association of serum *γ*-GTP and serum-carotenoid concentration with alcohol intake level has not been thoroughly studied in subjects without liver dysfunction. Therefore, in this study, we tested the hypothesis that serum-carotenoid concentration would be inversely associated with alcohol-induced oxidative stress within normal liver function. We evaluated the association of serum *γ*-GTP as a marker of oxidative stress induced by alcohol consumption and serum carotenoids: i.e., lutein, lycopene, *α*-carotene, *β*-carotene, *β*-cryptoxanthin, and zeaxanthin stratified by alcohol intake level in Japanese men and women within normal liver function.

## METHODS

In the present study, we used data derived from health examination of inhabitants aged from 30 to 70 years performed in the town of Mikkabi in Shizuoka Prefecture, Japan in April and May, 2003. A total of 1,979 males and females were subjects for the health examination. As results, 1,448 participants (73.2% of total subjects) had received the health examination. We recruited participants for this study and informed consent was obtained from 886 subjects (302 males and 584 females). The response rate was 61.2%. This study was approved by the ethics committee of the National Institute of Fruit Tree Science and the Hamamatsu University School of Medicine.

In this report, the following subjects were excluded from the data analysis: (1) those for whose serum aspartate aminotransferase (AST) and/or alanine aminotransferase (ALT) concentrations were defined as abnormalities (51+ IU/L and 46+ IU/L were used for cutoff values of high AST and ALT, respectively); (2) those with infected hepatic B or C virus or those who reported a history of liver diseases, in a self-administered questionnaire; and (3) those for whose blood samples for serum-carotenoid analysis were not collected. As a result, a total of 266 men and 557 women were included in this analysis.

A self-administered questionnaire was used to collect information about life-style characteristics, including tobacco use (current smoker, ex-smoker, or non-smoker), exercise habits (weekly participation), and dietary habits. Diet was assessed with a validated simple food-frequency questionnaire developed especially for the Japanese,^17^^,^^18^ and information about alcohol consumption and the daily intake of nutrients was obtained. In the part of the questionnaire dealing with food frequency, subjects were asked about their consumption of various types of alcoholic beverages (*sake*, beer, whisky, and others) for the previous month; daily ethanol consumption was then estimated from the frequency and volume of each alcoholic beverage consumed with the ethanol content of the corresponding beverage. The daily intakes of nutrients were estimated from the food intake frequencies per month with either standard portion size (for most of foods) or subject-specified usual portion size (for rice, breads, and alcoholic and non-alcoholic beverages). The total energy intake (including or excluding energy intake from ethanol) of all subjects was used in this report.

Height and body weight were measured by trained public health nurses. The body mass index (BMI) was calculated as the body weight (kg) divided by the height (m) squared. Blood pressure was measured using an automated sphygmomanometer Model BP-103iII (Nihon Colin, Inc., Aichi, Japan).

Blood samples were obtained in the morning after the subjects had fasted overnight. Serum was separated from blood cells by centrifugation and stored at -80°C until serum-carotenoid analyses. The concentrations of serum carotenoids, lutein, lycopene, *α*-carotene, *β*-carotene, *β*-cryptoxanthin, and zeaxanthin were analyzed by reverse-phase high-performance liquid chromatography (HPLC) using *β*-apo-8′-carotenal as an internal standard at the laboratory of Public Health and Environmental Chemistry, Kyoto Biseibutsu Kenkyusho (Kyoto, Japan). After storage for no longer than a year, serum samples were mixed with H_2_O and ethanol containing *β*-apo-8′-carotenal and extracted into hexane. The organic layer was removed, evaporated to dryness at room temperature, resolved in chloroform: ethanol (1:19), and transferred to a microvial for automatic injection. The HPLC system was Model HP-1100 (Hewlett-Packard, Toronto, Canada) fitted with a 201TP54 reverse-phase C18 column (Grace/Vydac, CA, USA). Six carotenoids were monitored at 480 nm in the HPLC system. The mobile phase component was methanol: tetrahydrofuran: H_2_O (94: 5: 1), and the flow rate of the phase was 0.8 ml/min. The peaks of the six carotenoids were identified by the retention time and quantified using standard curves of authentic lutein, lycopene, *α*-carotene, *β*-carotene, *β*-cryptoxanthin, and zeaxanthin (FUNAKOSHI, Tokyo, Japan). Quality control of the measurement of serum carotenoid concentration was maintained using pooled serum samples from five subjects as described previously.^19^

Serum total cholesterol, triacylglycerols, AST, ALT, and *γ*-GTP concentrations were measured by an auto-analyzer using commercial kits (Determiner TC-II C for serum total cholesterol, Kyowa-Medics, Inc., Tokyo, Japan; Determiner TG-II C for serum triacylglycerols, Kyowa-Medics, Inc., Tokyo, Japan; Quick-auto neo AST JS R for serum AST, SHINO-TEST, Inc., Tokyo, Japan; Quick-auto neo ALT JS R for serum ALT, SHINO-TEST, Inc., Tokyo, Japan; Quick-auto neo G-GT JS R for serum *γ*-GTP, SHINO-TEST, Inc., Tokyo, Japan). Plasma samples were obtained in sampling vials containing sodium fluoride. Fasting plasma glucose (FPG) was measured by an auto-analyzer (Glucoroder MAX, SHINO-TEST, Inc., Tokyo, Japan). All blood measurements except for serum carotenoid concentrations were conducted at the laboratory of Seirei Preventive Health-Care Center (Shizuoka, Japan).

The subjects were divided into three groups stratified by ethanol intake levels defined as non-drinker (less than 1 g of ethanol daily), light-drinker (1-25 g of ethanol daily), and moderate and heavy drinkers (25+ g of ethanol daily). In female subjects, the rate of moderate and heavy drinkers was only 1.1%. Therefore, a total of 266 males were included in further data analysis. Serum carotenoid concentrations, FPG, AST, ALT, *γ*-GTP, and triacylglycerols were skewed toward the higher concentrations. These values were log_e_ (natural) transformed to improve the normality of their distribution. Analysis of covariance adjusted for age followed by Bonferroni multiple comparisons were used to test between-group differences in ethanol intake levels. All variables were presented as their original scale. Associations among continuous variables across three groups of ethanol intake level were assessed by tests for linear trend using linear regression.

The subjects were further subcategorized into three groups according to the tertile of serum-carotenoid concentrations after stratified by ethanol intake levels. The multivariate-adjusted geometric mean and 95% confidence interval of the serum *γ*-GTP concentrations by tertiles of the serum-carotenoid concentration were calculated after adjusting for age, BMI, total cholesterol, triacylglycerols, current tobacco use, and exercise habits using analysis of covariance. In moderate and heavy drinkers, ethanol intakes of the highest tertiles of serum concentrations of lycopene and *β*-carotene were significantly lower than those in the lowest tertiles; therefore, in the group of moderate and heavy drinkers, ethanol intake was further adjusted. Differences of multivariate adjusted geometric mean of serum *γ*-GTP concentrations among each tertiles of serum carotenoid concentration were tested by Bonferroni multiple comparison.

The detection limit for serum lycopene concentration for the method used in the study was 0.04 *μ*g/rnL (0.07 *μ*mol/L), and the values below the limit of detection of the assay were marked as zero in the analysis. Data were analyzed using SPSS^®^ 12.0J for Windows (SPSS Inc., Chicago, USA).

## RESULTS

Table 1 shows the characteristics of the study subjects stratified by ethanol intake level. The serum *γ*-GTP, AST, and systolic blood pressure in moderate and heavy drinkers were significantly higher than those in non-drinker. Although total energy intake, including that from ethanol, in moderate and heavy drinkers was significantly higher than that in non-drinker, the total energy intake, excluding that from ethanol, was not different among the three groups. Serum lycopene, *α*-carotene, *β*-carotene, and *β*-cryptoxanthin concentrations in moderate and heavy drinkers were significantly lower than those in non-drinker.

**Table 1.** Characteristics of study subject stratified by ethanol intake level.

	Non-drinker (less than 1 g/day)		Light-drinker (1-25 g/day)			Moderate and heavy drinkers (25+ g/day)			P for trend
n	54		102			110			
Age (year)	57.5	(9.5)	55.5	(10.3)		57.3	(9.5)		0.447
Body mass index (kg/m^2^)	23.4	(3.1)	23.5	(2.8)		23.3	(2.6)		0.768
Systolic blood pressure (mmHg)	128.4	(20.1)	128.4	(16.9)		137.3	(20.6)	*	< 0.001
Serum total cholesterol (mmol/L)	5.1	(0.7)	5.3	(0.7)		5.3	(0.9)		0.475
Serum triacylglycerols (mmol/L)	1.2	( 1.0 - 1.3)	1.2	( 1.1 - 1.3)		1.3	( 1.2 - 1.4)		0.173
Fasting plasma glucose (mmol/L)	5.3	( 5.1 - 5.6)	5.5	( 5.3 - 5.7)		5.7	( 5.5 - 6.0)		0.051
*γ*-GTP (IU/L)	23.1	(20.6 - 26.0)	28.4	(25.8 - 31.3)		45.2	(40.1 - 50.9)	***	< 0.001
AST (IU/L)	20.2	(19.1 - 21.4)	20.1	(19.2 - 21.0)		22.5	(21.5 - 23.5)	*	< 0.001
ALT (IU/L)	19.5	(17.6 - 21.7)	18.7	(17.5 - 20.0)		20.2	(18.8 - 21.7)		0.205
High *γ*-GTP (%)**^†^**	1.9		6.9			30.9			
Total energy intake (kcal/day)									
including ethanol	1946.6	(453.7)	2151.1	(565.8)		2337.9	(567.2)	**	< 0.001
excluding ethanol	1945.9	(453.6)	2079.1	(562.9)		1991.4	(526.1)		0.682
Ethanol intake (g/day)	0.1	(0.2)	9.3	(6.8)	*	53.5	(30.7)	***	< 0.001
Current tobacco use (%)	31.5		25.5			38.2			
Habitual exercise (%)**^‡^**	9.3		18.8			30.0			
Serum carotenoid levels (*μ*mol/L)									
Lutein	0.53	(0.47 - 0.61)	0.51	(0.47 - 0.56)		0.58	(0.54 - 0.63)		0.041
Lycopene	0.24	(0.20 - 0.29)	0.22	(0.19 - 0.26)		0.16	(0.14 - 0.19)	**	< 0.001
*α*-Carotene	0.11	(0.10 - 0.13)	0.11	(0.10 - 0.12)		0.09	(0.08 - 0.10)	**	< 0.001
*β*-Carotene	0.53	(0.46 - 0.61)	0.45	(0.40 - 0.51)		0.33	(0.30 - 0.37)	***	< 0.001
*β*-Cryptoxanthin	1.43	(1.15 - 1.79)	1.23	(1.02 - 1.48)		0.92	(0.78 - 1.09)	**	0.002
Zeaxanthin	0.23	(0.21 - 0.26)	0.23	(0.21 - 0.24)		0.24	(0.23 - 0.25)		0.328

Data are mean (standard deviation), geometric mean (95% confidence interval), or percent.

† : 60+ IU/L of serum *γ*-GTP

‡ : 1+ times/week

*: P<0.05, ** :P<0.01, and ***: P<0.001 vs non-drinker by analysis of covariance adjusted for age followed by Bonferroni multiple comparison test.

*γ*-GTP: gamma-glutamyltransferase, AST: aspartate aminotransferase, ALT: alanine aminotransferase.

The multivariate-adjusted geometric means of serum *γ*-GTP concentrations associated with the tertiles of each serum-carotenoid concentration stratified by ethanol intake level are shown in Table 2. In non-drinker, adjusted means of serum *γ*-GTP concentrations were not different among tertiles of all serum carotenoid concentrations. In light-drinker, the adjusted means of serum *γ*-GTP were slightly low in accordance with tertiles of serum *β*-carotene and *β*-cryptoxanthin concentration but the group difference was not statistically significant. In moderate and heavy drinkers, adjusted means of serum *γ*-GTP were significantly low in accordance with the tertiles of serum lycopene, *α*-carotene, *β*-carotene, and *β*-cryptoxanthin.

**Table 2.** Multivariated-adjusted geometric means of serum gamma-glutamyltransferase (*γ*-GTP) concentration by tertiles of serum-carotenoid concentrations stratified by ethanol intake levels.

Senim carotenoid		Non-drinker (less than 1 g/day)			Light-drinker (1-25 g/day)			Moderate and heavy drinkers (25+ g/day)		
		
		n	Mean and range of senim carotenoid (*μ*mol/L)	Adjusted mean and 95% CI of senim *γ*-GTP(IU/L)^†^	n	Mean and range of senim carotenoid (*μ*mol/L)	Adjusted mean and 95% CI of senim *γ*-GTP(IU/L)^†^	n	Mean and range of serum carotenoid (*μ*mol/L)	Adjusted mean and 95% CI of serum *γ*-GTP(IU/L)^‡^
Lutein	Lowest	18	0.32 (0.19 - 0.42)	25.6 (20.9 - 31.3)	30	0.32 (0.16 - 0.42)	29.3 (24.6 - 34.8)	37	0.39 (0.19 - 0.49)	48.8 (40.2 - 59.2)
	Middle	17	0.53 (0.44 - 0.63)	25.5 (20.9 - 31.0)	39	0.51 (0.44 - 0.58)	26.5 (22.8 - 30.7)	39	0.58 (0.51 - 0.67)	42.6 (35.2 - 51.4)
	Highest	19	0.87 (0.67 - 1.79)	19.3 (16.0 - 23.4)	33	0.80 (0.60 - 1.85)	30.3 (25.5 - 36.0)	34	0.91 (0.69 - 1.60)	44.5 (36.2 - 54.9)

Lycopene	Lowest	18	0.11 (0.06 - 0.17)	25.7 (20.9 - 31.4)	34	0.10 (0.06 - 0.17)	32.2 (27.5 - 37.8)	40	0.07 (0.06 - 0.09)	54.9 (45.7 - 65.9)
	Middle	19	0.25 (0.19 - 0.34)	21.4 (17.7 - 25.9)	34	0.24 (0.19 - 0.32)	24.6 (21.0 - 28.8)	33	0.17 (0.11 - 0.28)	43.8 (36.2 - 53.2)
	Highest	17	0.54 (0.37 - 0.91)	22.7 (18.5 - 27.9)	34	0.48 (0.34 - 0.84)	29.0 (24.8 - 33.9)	37	0.41 (0.30 - 1.06)	37.6 (31.0 - 45.7) *

*α*-Carotene	Lowest	16	0.07 (0.06 - 0.07)	24.3 (19.6 - 30.0)	28	0.06 (0.04 - 0.07)	28.9 (24.0 - 34.7)	47	0.06 (0.04 - 0.07)	54.4 (46.0 - 64.3)
	Middle	22	0.11 (0.09 - 0.13)	24.4 (20.4 - 29.2)	40	0.10 (0.09 - 0.11)	29.1 (25.1 - 33.8)	28	0.09 (0.09 - 0.09)	40.8 (33.0 - 50.5)
	Highest	16	0.20 (0.15 - 0.52)	20.5 (16.4 - 25.8)	34	0.18 (0.13 - 0.73)	27.4 (23.2 - 32.4)	35	0.15 (0.11 - 0.32)	38.2 (31.3 - 46.6) *

*β*-Carotene	Lowest	17	0.30 (0.22 - 0.39)	23.5 (18.9 - 29.3)	36	0.25 (0.11 - 0.34)	34.3 (29.3 - 40.2)	32	0.18 (0.09 - 0.24)	63.7 (51.7 - 78.5)
	Middle	19	0.48 (0.41 - 0.60)	25.3 (20.7 - 30.8)	32	0.44 (0.35 - 0.56)	26.8 (22.8 - 31.4)	41	0.31 (0.26 - 0.37)	44.7 (37.9 - 52.8) *
	Highest	18	0.98 (0.63 - 1.88)	20.8 (16.7 - 25.7)	34	0.87 (0.58 - 1.96)	24.6 (20.9 - 29.1)	37	0.62 (0.39 - 1.30)	33.9 (28.1 - 41.1) **

*β*-Cryptoxanthin	Lowest	17	0.60 (0.18 - 0.94)	29.7 (23.4 - 37.6)	34	0.41 (0.14 - 0.89)	32.7 (27.7 - 38.7)	36	0.33 (0.14 - 0.51)	58.8 (48.7 - 70.8)
	Middle	19	1.32 (0.96 - 2.13)	20.2 (16.6 - 24.5)	34	1.33 (0.92 - 2.01)	27.9 (23.8 - 32.8)	37	0.92 (0.52 - 1.47)	48.6 (40.6 - 58.1)
	Highest	18	3.56 (2.19 - 6.66)	21.2 (17.3 - 25.8)	34	3.37 (2.12 - 6.57)	25.2 (21.4 - 29.8)	37	2.48 (1.50 - 8.19)	32.5 (27.2 - 39.0) **

Zeaxanthin	Lowest	20	0.17 (0.09 - 0.21)	26.3 (21.8 - 31.7)	38	0.17 (0.12 - 0.19)	29.3 (25.0 - 34.3)	42	0.18 (0.11 - 0.21)	50.4 (42.2 - 60.3)
	Middle	15	0.24 (0.23 - 0.25)	21.2 (17.2 - 26.1)	27	0.23 (0.21 - 0.25)	26.2 (21.9 - 31.3)	31	0.24 (0.23 - 0.26)	47.7 (39.1 - 58.3)
	Highest	19	0.33 (0.26 - 0.54)	21.7 (17.9 - 26.3)	37	0.31 (0.26 - 0.47)	29.4 (25.0 - 34.5)	37	0.33 (0.28 - 0.62)	38.1 (31.6 - 46.0)

† : Adjustments were made for age, body mass index, total cholesterol, triacylglycerols, current smoking, and habitual exercise.

‡ : Adjusted for age, body mass index, total cholesterol, triacylglycerols, current smoking, habitual exercise, and ethanol intake.

* : P<0.05, **: P<0.001 vs the lowest carotenoid tertile in Bonferroni multiple comparison test.

## DISCUSSION

This study aimed to investigate whether serum-carotenoid concentration would be inversely associated with alcohol-induced oxidative stress within normal liver function. As results, we found the inverse associations between serum lycopene, *α*-carotene, *β*-carotene, and *β*-cryptoxanthin with serum *γ*-GTP were more evident with increased alcohol consumption.

A recent epidemiologic study has reported the inverse association of serum *γ*-GTP with serum-carotenoid concentrations both cross-sectionally and longitudinally.^13^ In this study, the inverse associations between total serum carotenoid concentration and serum *γ*-GTP were examined among subgroups including race, sex, BMI levels, tobacco use, and vitamin supplement usage. With regard to alcohol consumption, although inverse associations between total serum carotenoid concentration and serum *γ*-GTP were found in drinkers, they were not found in non-drinkers.^13^ However, the associations of the serum-carotenoid concentration and serum *γ*-GTP with ethanol intake were not discussed in detail according to the stratification of the ethanol intake level. Furthermore, the associations of serum concentration of each carotenoid with serum *γ*-GTP were not explained in detail. In our study, the daily ethanol consumption of study subjects was estimated precisely from the data obtained in the questionnaire concerning food frequency. Therefore, these data make it possible to evaluate the detailed association of serum-carotenoid concentrations and serum *γ*-GTP with alcohol intake level. As a result, we found that the inverse associations of the serum-carotenoid concentrations and serum *γ*-GTP were more evident in moderate and heavy drinkers than in light-drinker.

Gamma-GTP exists widely in various tissues, especially in the liver and kidneys, and it catalyses the transfer of a *γ*-glutamyl group from *γ*-glutamyl peptides to other peptides, thereby providing a supply of constituent amino acids for uptake and reutilisation in intracellular glutathione synthesis.^20^^-^^22^ In a normal metabolism, this enzyme plays an important role in antioxidant defense systems on a cellular level. The absorbed ethanol is oxidised to acetaldehyde by acetaldehyde dehydrogenase in mitochondria. In habitual drinkers, the microsomal ethanol-oxidising system is increased by enzyme induction and is also responsible for the production of acetaldehyde.^1^^-^^3^ The generation of high concentrations of free radical species during the metabolism of alcohol may exceed the capacity of the antioxidant defence mechanisms and cause the development of liver dysfunction.

Recent epidemiologic studies have shown that the serum concentration of carotenoid is well correlated with the intake of fruits and vegetables rich in carotenoids.^23^^-^^25^ Furthermore, high correlation coefficients of dietary intake of carotenoids from fruit and vegetables with serum levels have been reported.^26^^,^^27^ It has been reported that ingested carotenoids from foods also exist in several human organs.^28^^-^^31^ Carotenoids are mainly accumulated in the liver and combined into lipoprotein for release into the blood circulation. It is conceivable that ingested carotenoids could participate in an antioxidant defense system when present in high concentrations of free radical species during the metabolism of alcohol in the liver.

There are several limitations in the present study. Firstly, the data obtained here consisted of cross-sectional analyses. Therefore, our data limits inferences on temporality and causation. Secondly, we could not evaluate the association of serum carotenoid concentrations and serum *γ*-GTP in heavy-drinker (50+ g of ethanol daily) because the number of subjects in the heavy-drinker (n=45) was too small for further subgroup analyses. Thirdly, we have no data about serum levels of other antioxidant vitamins such as vitamin C or *α*-tocopherol.

In conclusion, the inverse associations between serum lycopene, *α*-carotene, *β*-carotene, and *β*-cryptoxanthin with serum *γ*-GTP were observed in moderate and heavy drinkers. These results suggest that carotenoids may have an antioxidant effect against alcohol-induced oxidative stress. To determine whether serum carotenoids are effective against alcohol-related oxidative stress, further cohort studies or intervention studies will be required.
